# High-speed roll-to-roll manufacturing of graphene using a concentric tube CVD reactor

**DOI:** 10.1038/srep10257

**Published:** 2015-05-21

**Authors:** Erik S. Polsen, Daniel Q. McNerny, B. Viswanath, Sebastian W. Pattinson, A. John Hart

**Affiliations:** 1 Department of Mechanical Engineering, University of Michigan, 2350 Hayward St., Ann Arbor, MI 48109, USA; 2 Department of Mechanical Engineering and Laboratory for Manufacturing and Productivity, Massachusetts Institute of Technology, 77 Massachusetts Avenue, Cambridge, MA 02139, USA

## Abstract

We present the design of a concentric tube (CT) reactor for roll-to-roll chemical vapor deposition (CVD) on flexible substrates, and its application to continuous production of graphene on copper foil. In the CTCVD reactor, the thin foil substrate is helically wrapped around the inner tube, and translates through the gap between the concentric tubes. We use a bench-scale prototype machine to synthesize graphene on copper substrates at translation speeds varying from 25 mm/min to 500 mm/min, and investigate the influence of process parameters on the uniformity and coverage of graphene on a continuously moving foil. At lower speeds, high-quality monolayer graphene is formed; at higher speeds, rapid nucleation of small graphene domains is observed, yet coalescence is prevented by the limited residence time in the CTCVD system. We show that a smooth isothermal transition between the reducing and carbon-containing atmospheres, enabled by injection of the carbon feedstock via radial holes in the inner tube, is essential to high-quality roll-to-roll graphene CVD. We discuss how the foil quality and microstructure limit the uniformity of graphene over macroscopic dimensions. We conclude by discussing means of scaling and reconfiguring the CTCVD design based on general requirements for 2-D materials manufacturing.

The integration of two-dimensional (2-D) materials with applications that demand cost-effective large-area production requires understanding of how lab-scale synthesis methods can be translated to continuous manufacturing processes. For thin films of graphene, such promising applications include transparent electrodes for displays and photovoltaics, high-performance filtration membranes, and thermal imagers^1^,^2^,^3^,^4^,^5^,^6^,^7^. Direct synthesis of graphene on substrates by chemical vapor deposition (CVD) has emerged as a highly attractive technique for these applications because of its compatibility with thin film processing tools, and its potential scalability to large areas^1^. As a result of continued research efforts, the electrical transport of graphene synthesized by CVD on substrates is approaching that of exfoliated graphene, and a growing portfolio of CVD recipes can be applied to substrates of increasing size (centimeter to wafer scale) and diversity (*e.g.*, metallic thin films deposited on Si and quartz, in addition to metal foils)^2^,^8^,^9^,^10^,^11^,^12^.

Several systems and methods for roll-to-roll (R2R) graphene production have been presented in the academic literature^13^,^14^,^15^,^16^,^17^. Early on, Hesjedal and colleagues used a modified tube furnace for R2R production of multi-layer graphene on Cu foil (25 μm thick, 1 m length) at 1–40 cm/min^14^. Yamada and colleagues presented a custom microwave plasma CVD system and reported complete coverage of multilayer graphene at a feed rate of 30 cm/min using Cu foil with 294 mm width^15^. While the plasma-enhanced process enabled low temperature growth (>400 °C), this also limited the graphene quality and domain size. More recently, Kobayashi and colleagues produced high quality, predominantly single-layer graphene on Cu foil (230 mm wide, 36 μm thick) at 10 cm/min using a R2R CVD system that resistively heated the Cu foil fed between two electrode rollers^17^. Following subsequent transfer, graphene coverage of 89–98% was reported on the final substrate which was a polyethylene terephthalate (PET) film. In parallel with these efforts, notable progress has been made on batch-style CVD growth. In 2010, Bae *et al.* produced uniform graphene films on 30" diagonal Cu foils that were wrapped around a 7.5” diameter quartz tube placed for static processing inside an 8” diameter quartz tube within a tube furnace. The graphene films were subsequently transferred to PET following a wet chemical etch of the Cu^13^. A similar technique was used by Vlassiouk *et al.* to produce 40" diagonal films of graphene that were subsequently transferred to PET^16^.

Despite these achievements, it is still necessary to advance continuous production of 2D materials to reflect a rigorous understanding of the underlying process physics, and to enable high-quality layer-controlled production at a high rate. For graphene in particular, the design of the R2R CVD system is critical to establish such understanding, and design principles that should be captured in an effective system include: thermal and fluidic uniformity over the substrate; efficient mixing and use of the feedstock gases; sealed and controlled gas atmospheres and thermal zones (*e.g.*, as seen in carbon fiber production)^18^,^19^,^20^; and throughput that is compatible with upstream and downstream processes (*e.g.*, integration with patterning operations)^15^,^21^. Also, graphene growth using CVD requires sequential heating in an inert or reducing atmosphere followed by hydrocarbon exposure, and substrate handling and the transitions between zones must consider this requirement^22^. Practically, there also exists a need to understand the dependence of key graphene characteristics on the multi-dimensional parameter space of a continuous process (*e.g.*, temperature, pressure, atmosphere composition, feed rate, quality) similar to parametric studies performed for batch-scale graphene growth using static reactor conditions^22^,^23^,^24^,^25^. This would, in turn, enable engineering of graphene characteristics (*e.g.*, number of layers, domain size, quality) to meet both application-oriented needs and production specifications (*e.g.*, cost, rate).

We present a new reactor design for R2R CVD of 2-D materials on flexible substrates, and using a benchtop prototype of this reactor, we study R2R production of graphene on metal foils. The reactor has a concentric tube geometry, which achieves several desirable features for R2R CVD, including thermal and fluidic uniformity over the substrate due to the small gap, a rapid isothermal transition between the two internal atmospheres *via* downstream injection of the hydrocarbon precursor, and modularity due to its circular geometry. Using the concentric tube system, we find an inverse relationship between graphene film quality and production speed, which is governed by the nucleation and coalescence kinetics of graphene domains in combination with the residence time of the substrate. The downstream injection of the hydrocarbon into the CTCVD system yields roughly 2.7x and 1.8x increases in I_2D_/I_G_ and I_G_/I_D_ respectively, yet overall graphene quality is limited by the grain size and surface quality of the copper foil. Last, we study the influence of annealing time, reactor temperature, and cooling atmosphere, and find that additional annealing time (3 hours), increased reactor temperature (from 1000 °C to 1045 °C), and a He/H_2_ cooling atmosphere give a 1.9x, 1.4x and 1.9x improvement of I_2D_/I_G_, respectively.

## Concentric Tube Reactor

**Figure 1:**
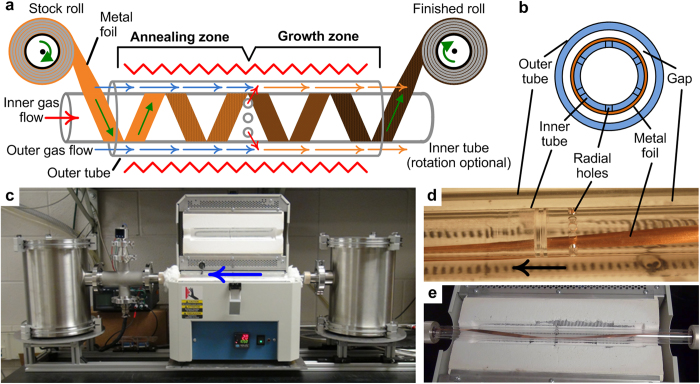
Concentric tube CVD (CTCVD) system configured for R2R graphene growth on Cu foil. **a**) System schematic showing the helical feed path (left to right), sequential treatment zones, and internal gas injection holes. **b**) Cross-section view of the concentric tube arrangement. **c**) Bench-scale prototype of the CTCVD system (setup where processing is right to left), with rails for alignment with tube furnace. **d**) Close-up of e), showing the gas injection holes used to supply the hydrocarbon gas to the downstream treatment zone. **e**) Top view of a Cu foil substrate wrapped through the system.

In the concentric tube (CT) CVD reactor design (Fig. 1a–b), the substrate continuously translates in a helical path, as it is wrapped onto the surface of a quartz tube placed concentrically within another quartz tube. The heated reactor volume is therefore defined by the annular gap between the tubes and the length over which the system is heated. Compared to a single tube reactor design with equivalent outer diameter, the rationale for the CTCVD configuration is to reduce the volume of gas required for processing, establish flow uniformity *via* the small gap between the tubes, and enable the size of the treatment zone to be adjusted without changing the flow profile over the substrate. The prototype CTCVD system is built using a standard tube furnace (Lindberg Blue M Mini-Mite, single 30cm long heated zone), and the end chambers contain web-handling mechanisms built largely using commercial-off-the-shelf components (Fig. 1c–e). The use of tubes with circular cross section is desirable for low-pressure operation and sealing using conventional vacuum components, while the annular reactor geometry captures the geometric advantage of a thin cross-section.

**Figure 2:**
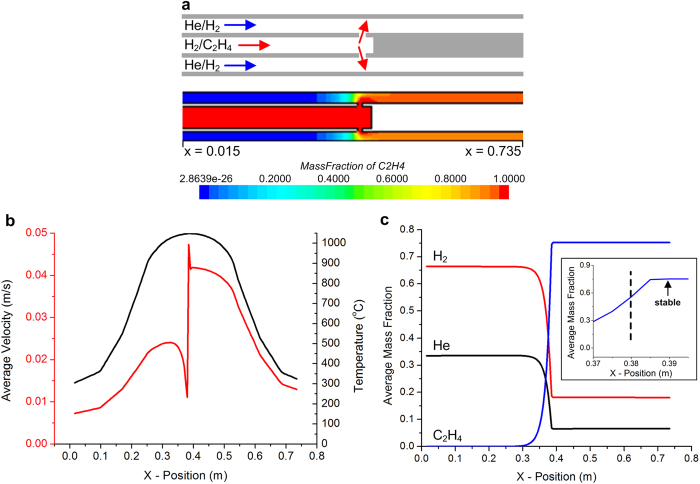
Computational fluid dynamics (CFD) model of gas flow within the CTCVD reactor. **a**) Cross-section diagram of the flow paths within the concentric tube arrangement and colormap of C_2_H_4_ mass fraction during steady operation. **b**) Profiles of average gas velocity in the gap between the tubes and average temperature versus position along the flow direction. **c**) Mass fraction of He, H_2_, and C_2_H_4_ along the flow direction, showing the abrupt change upon injection of C_2_H_4_ through the inner tube, and the rapid stabilization <1 cm downstream of this point (x = 0.38 m). Data is from CFD simulations run at a system pressure of 760 torr.

A further attractive feature of the CTCVD design is the ability to create two sequential treatment zones (Fig. 2a) *via* the injection of the precursor gas through the inner tube. For example, a first gas mixture such as He/H_2_ is supplied to the upstream chamber, and flows into the gap between the tubes; and a second gas mixture including the precursor is directly fed to the inner tube. The inner tube is custom-made with radial holes and a blockage adjacent to the holes; these direct the second gas mixture to enter the annular gap, creating the second gas treatment zone without changing the local temperature profile. The flow dynamics in the CTCVD system with downstream injection were studied using computational fluid dynamics (CFD) simulations, at system pressures of 10^–3^ and 760 torr. The mixing of C_2_H_4_ upon injection from the inner tube is visualized in Fig. 2a, where the tube gap is 4.5 mm. The injection of the precursor into the annular gap increases the average gas velocity from 0.024 m/s to 0.048 m/s (Fig. 2b), and the velocity and chemistry of the gas mixture are stable to within 99% of their final values at a point 10 mm downstream of the injection holes (Fig. 2b,c). The rapid deceleration and acceleration of the gases at the injection location is due to the gas flow through the inner tube holes impinging on the inner wall of the outer tube. The injection causes slight backward flow of the carbon precursor due to diffusive mixing and impingement of the gas on the inner wall of the outer tube, but a net forward velocity is maintained.

Additionally, the design of the CTCVD system enables a seamless thermal gradient (Fig. 2b) across the two treatment zones because the gas injected downstream is preheated within the inner tube. To demonstrate this, the gas temperature in the CTCVD setup was calculated based on the temperature profile along the central axis of the empty furnace. The continuous thermal profile is an important consideration because it is known that hydrocarbon introduction at below the desired graphene growth temperature can inhibit graphene formation or cause an undesirably greater nucleation density of smaller graphene domains, as described by Robinson and Robins^22^,^26^.

## Results

### Graphene synthesis

Using the CTCVD system, we first chose to investigate the relationship between the process velocity and the characteristics of graphene produced on Cu foil (see Methods). Experiments were performed at translation speeds between 25 mm/min and 500 mm/min, under identical temperature and gas flow conditions (see Methods). We performed independent tests at each speed (*i.e.*, where the system was fully stopped and reloaded between tests) and a continuous run where the velocity was adjusted as the substrate was fed through the CTCVD reactor In both cases, graphene was grown on 60 cm of foil at each velocity. No statistically relevant differences in the graphene produced were found when comparing the two experimentation methods.

**Figure 3:**
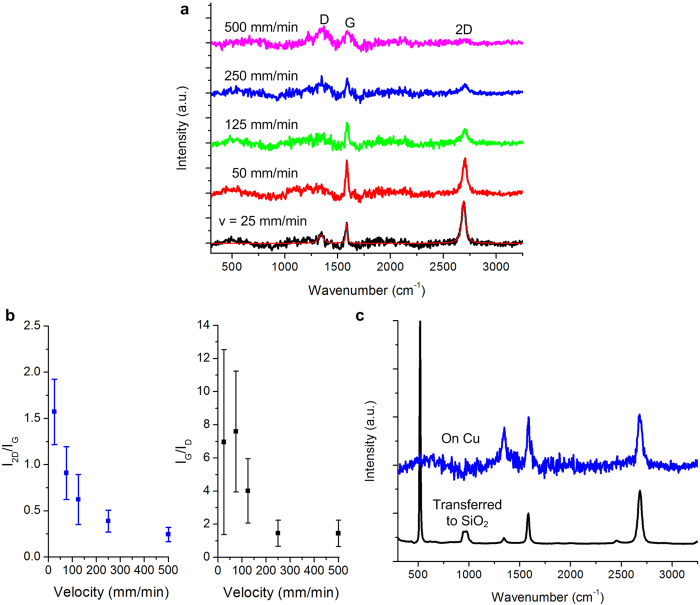
Influence of substrate velocity on roll-to-roll graphene synthesis on copper foil. **a**) Average Raman spectra for each of the five velocities tested, from 25 mm/min to 500 mm/min. A polynomial fit of the background signal was subtracted from the raw Raman spectra, resulting in the spectra shown. Additional post processing (see Methods) was applied to each spectrum prior to analysis of the peak intensities. An example post-processed spectrum is overlaid on the 25 mm/min background subtracted data (red). **b**) Average I_2D_/I_G_ and I_G_/I_D_ values versus substrate velocity. **c**) Comparison of Raman spectra (25 mm/min) before and after transfer to SiO_2_ (see Methods).

In Fig. 3a, we show a typical Raman spectrum for each speed, based on data collected at 27 locations across each sample. Characteristic D, G and 2D peaks were observed at all speeds, and the D peak intensity increased while the G peak intensity decreased with increasing translation speed. Accordingly, the I_2D_/I_G_ ratio decreases as the velocity increases (Fig. 3b), implying either an increasing number of graphene layers or an increase in the density of graphene edges. Two-dimensional Raman maps of the same substrates also indicate that the I_2D_/I_G_ ratio trend with velocity is consistent across larger sample areas (see Supporting Information, Fig. S1). A similar inverse relationship is observed between the I_G_/I_D_ ratio and substrate velocity (Fig. 3b), indicating an increase in defects or free edges^27^. Additionally, the average full width at half-maximum (FWHM) values of the 2D peaks increase with speed from 36 to 79 cm^−1^ (see Supporting Information, Fig. S2), which may indicate an increase in the number of graphene layers (*i.e.*, from monolayer to multi-layer graphene), or increasing edge defect density at higher substrate velocity^25^,^28^.

After transfer to SiO_2_, samples processed at 25 mm/min show a clean spectrum consistent with high-quality monolayer graphene (Fig. 3c). The SiO_2_ substrate yields a Raman spectrum with a much greater signal to noise ratio, and the post-transfer spectra represent the apparent best result for graphene produced in the present study using the CTCVD system. The transfer of graphene also resulted in a noticeable change in the relative peak intensities, where the I_2D_/I_G_ ratio increased from 1.1 on Cu to 1.5 on SiO_2_, and the I_G_/I_D_ ratio greatly improved from 1.5 on Cu to 6.1 on SiO_2_. We considered whether this difference arose from graphene damage by the laser on Cu; however, the Raman intensities did not change during prolonged (>2 min) exposures, and repeated interrogation of the same spots resulted in the same Raman spectrum as the first collection. Nevertheless, to avoid variation in results due to the transfer process itself, the following results are presented directly on the Cu substrate.

**Figure 4:**
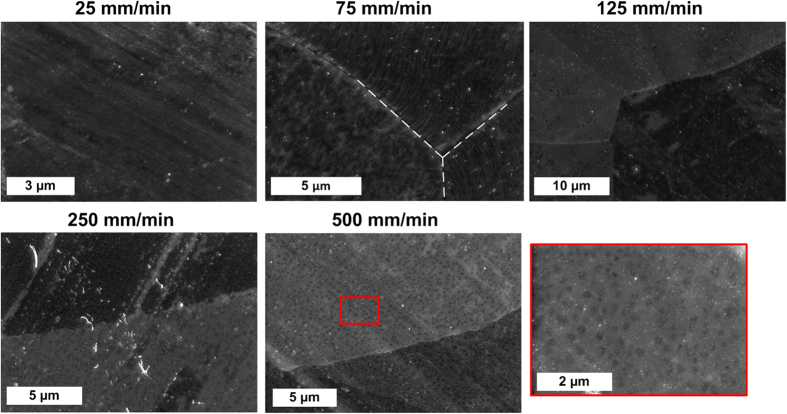
Influence of substrate velocity on graphene coverage, as shown by SEM images after CVD treatment of Cu. The large areas of similar contrast indicate Cu grains (with boundary indicated by the dashed lines in the 75 mm/min image), and the local dark regions within the individual grains correspond to graphene. Nanoscale domains of graphene are formed at short growth times (*i.e.*, as observed for high substrate velocity, 500 mm/min) and coalesce to form larger domains for longer growth times (*i.e.*, at lower substrate velocity). Prominent diagonal grooves in the 25, 250 and 500 mm/min images are due to the surface processing of the foil. High-magnification image of 500 mm/min sample most clearly shows isolated domains within a Cu grain.

High-resolution SEM imaging on Cu was also used to examine the uniformity of graphene coverage at different velocities (Fig. 4). Judging by the surface contrast at low accelerating voltage, we find near complete graphene coverage at low substrate velocities (*e.g.*, 2  mm/min), and isolated nanoscale graphene domains at high substrate velocities (*e.g.*, 500 mm/min). This agrees with previous studies that found graphene growth begins by formation of nanoscale domains at nucleation sites on Cu, which can coalesce given sufficient time^29^,^30^. The inverse relationship between graphene coverage and translation speed is also consistent with the Raman spectra (Fig. 3a). A high nucleation density could be conducive to high-speed growth if the graphene domains have a similar orientation and are able to coalesce into a single domain; however, as discussed later, the limited size and varied orientation of Cu grains presents a practical limit to production quality.

We also conclude that the inverse relationship between substrate velocity and I_2D_/I_G_ ratio is driven by edge defects rather than the number of graphene layers. In the case of uniform coverage, an increase in the number of graphene layers causes the 2D peak to broaden and shift to higher frequency. However, in the case of small graphene domains, the 2D peak is relatively much broader due to edge defects and orientation mismatches between domains. In the present study, as the translation speed increases and the coverage of graphene becomes less continuous, we observe that the 2D peak broadens and the peak intensity decreases. Incidentally, 2D peak broadening relative to graphene films has been observed in graphene quantum dots (GQDs) having diameters of 5–35 nm, where the 2D peak broadened as the number of edge defects and relative GQD orientation mismatch to neighboring GQDs increased^31^. Also, we suspect that the apparent increase in graphene quality upon transfer to SiO_2_ (*i.e.*, the decrease of the D-peak intensity and the increase of the I_2D_/I_G_ ratio in Fig. 3d) was influenced by the loss of some of the nanoscale domains during the transfer process (see Methods and Supporting Information, Fig. S3), resulting in areas with fewer edge defects.

### Graphene variation

We next assessed the graphene uniformity by comparing Raman spectra taken across the width and length of each sample (see Supporting Information, Fig. S4a). Additionally, a long (4 m) Cu substrate was translated through the reactor at a rate of 75 mm/min (53  minutes total run time), and the Raman data is shown in Fig. S4b (see Supporting Information) for locations every 30 mm along the length of the foil. Although there is a significant variation in the I_2D_/I_G_ and I_G_/I_D_ values along the width and length of the sample (0.6–1.7 and 2.1–10.0, respectively), we attribute this to the polycrystalline nature of the Cu substrate, not the reactor design.

The kinetics of graphene nucleation and growth will depend on the orientation and surface conditions of each Cu grain^32^,^33^,^34^,^35^,^36^,^37^,^38^; therefore, in spite of the observed rapid nucleation, the limited residence time in the CTCVD system resulted in graphene coverage that varies significantly within individual Cu grains (noticeable in SEM images of 125 and 250 mm/min samples in Fig. 4). For example, by HRSEM we found that groups of adjacent grains showed micron sized graphene domains that covered 50–90% of the Cu surface (see Supporting Information, Fig. S5a). Raman spectroscopy and visible light microscopy data was also collected for a similar neighborhood on the foil. Contrast in optical microscopy can also be used to judge uniformity within each grain (see Supporting Information, Fig. S5b).

In addition, the surface of the Cu foil had striations and mechanically formed defects (*i.e.*, surface roughness, pits, indentations) as a result of the manufacturing process (see Supporting Information, Fig. S6). This topography undoubtedly influences the uniformity and coverage of graphene by influencing the nucleation site density and presenting mechanical barriers to lateral growth. Comparison of the survey spectra (see Supporting Information, Fig. S4b) with the average spectra of several Raman scans conducted within individual Cu grains supports the theory that the variation seen across the length of the sample is due to the defects in the substrate surface and the polycrystalline nature of the substrate (see Supporting Information, Fig. S5c).

**Figure 5:**
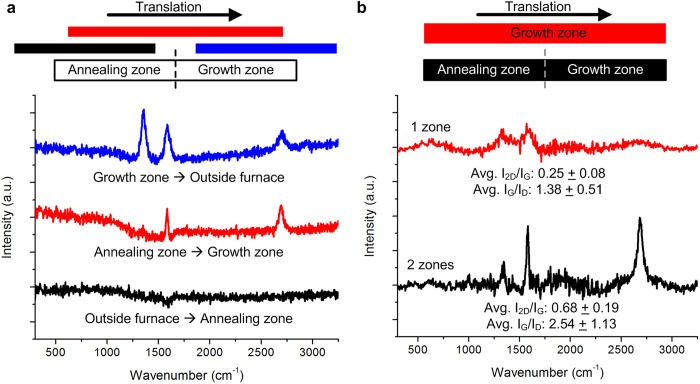
Benefit of continuous thermal processing with direct transition from a reducing to carbon-containing atmosphere. **a**) Raman spectra of Cu foil strips that were translated from upstream of the furnace to the annealing zone (black), from the annealing zone to the growth zone (red), and from the growth zone to the exit (downstream) of the furnace (blue). The sample sequentially exposed to the annealing and growth zones shows the best result, and growth does not occur in the annealing zone only. **b**) Comparison of Raman spectra for CTCVD processing with both zones to processing with the growth zone only.

To isolate the influence of each zone of the reactor, experiments were performed by moving the Cu foil from selected points spanning from upstream to downstream of the heated region, resulting in the Raman spectra shown in Fig. 5a. The portion of Cu foil that started upstream of the furnace and was stopped in the annealing zone did not have graphene present; the Cu that started in the annealing zone and ended in the growth zone exhibited high quality graphene; and the Cu location that started in the growth zone (*i.e.*, was heated while exposed to the carbon precursor) and ended downstream of the furnace had low quality graphene. Thus, we conclude that it is important to heat the Cu foil while exposing it to a non-carbon atmosphere, and to transition to the carbon atmosphere at elevated temperature. The benefit of downstream hydrocarbon injection is also illustrated in Fig. 5b. Here we compare results with a single zone CTCVD design (*i.e.*, H_2_/C_2_H_4_ injected in the annular gap from the input of the system, no downstream holes) and two treatment zones (normal CTCVD configuration). Downstream injection yields roughly 2.7x and 1.8x increases in I_2D_/I_G_ and I_G_/I_D_ respectively, relative to the single-zone design. The importance of an isothermal transition from a reducing atmosphere to a carbon-containing atmosphere was also highlighted in a recent study that used a diffusion barrier (Al_2_O_3_ on Ni) to prevent carbon exposure until the elevated temperature was reached in a single-zone system^39^.

**Figure 6:**
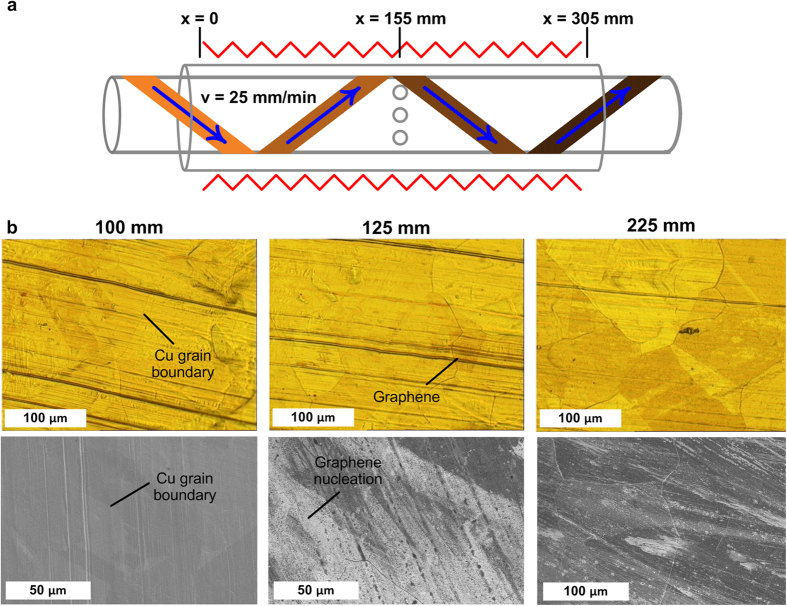
Analysis of sequential stages of R2R graphene growth: reduction of Cu, graphene nucleation, and graphene growth, as determined by the position of the foil along the CTCVD reactor. Translation of the Cu substrate was stopped to “freeze” the various stages along the length of the substrate while the system was cooled rapidly. **a**) Schematic of the CTCVD with axial positions highlighted relative to the beginning of the heated zone (the foil is translating left-to-right). **b**) Visible light microscopy (top) and SEM (bottom) images of a Cu substrate at various positions along the length of the CTCVD reactor (translated at 25 mm/min). Cu grain boundaries become visible as the oxide layer is reduced (x = 100 mm, left), followed by nucleation of graphene near the injection holes (x = 125 mm, middle), transitioning to near full graphene coverage prior to exiting the heated zone (x = 225 mm, right).

To further ascertain the substrate treatment kinetics within the CTCVD reactor, a Cu foil sample undergoing constant velocity processing was abruptly stopped and cooled rapidly by opening the furnace cover and applying a cold air stream across the reactor wall with a fan. Optical images and SEM images at marked locations on the substrate show the graphene coverage as a function of time in the reactor (Fig. 6a-b). Raman spectra of these locations are shown in the Supporting Information, Fig. S7. Emergence of visible grain boundaries upon annealing indicates that the surface oxide layer is reduced during H_2_ annealing at elevated temperature. At 25 mm/min, the exposure of the grain boundaries during the annealing of the Cu requires ~100 mm of travel (4 min residence time), and at ~125 mm (~25 mm upstream of the inner tube injection holes) we find that Cu grains begin to darken optically, and nanoscale graphene domains are found in the SEM images. This is also the first location along the length of the substrate that G, D and 2D peaks are observable on the Raman spectrum. As the foil progresses further through the reactor, maximum coverage of graphene is achieved between the 175 mm and 225 mm locations (as determined by Raman spectra line scans along the width of the foil where D, G and 2D peaks were always apparent, peak intensity ratios I_2D_/I_G_ and I_G_/I_D_ were maximized, and the coverage depicted in the SEM images), representing 120–240 seconds of exposure beyond the nucleation point. Moreover, we find some Cu grains become nearly fully covered within less than 45 seconds (at the end of a 250 mm/min run), compared to the much longer time to achieve maximum coverage (120–240 seconds) on the polycrystalline foil (See Supporting Information, Fig. S8). Improved crystallinity and surface conditions of the foil could therefore greatly increase the throughput and quality of the R2R CVD process.

### Process improvements

**Figure 7:**
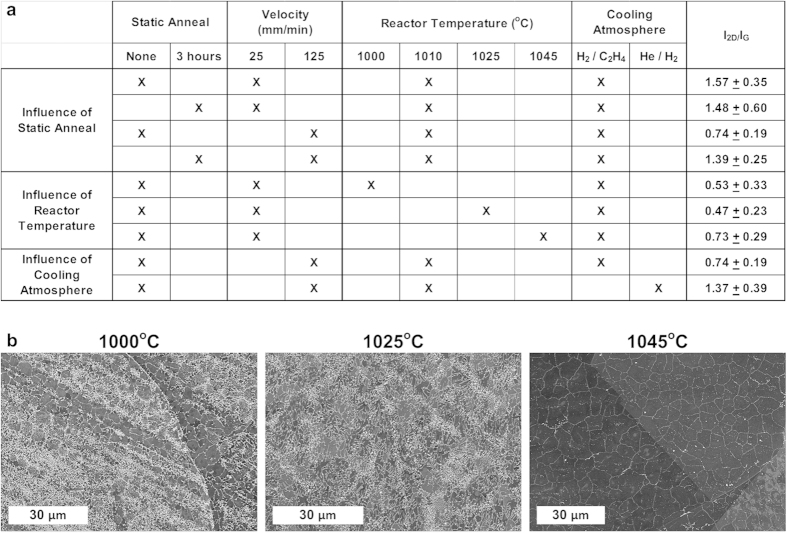
Improvement of graphene coverage and quality by exploring a matrix of process parameters using the CTCVD system, focusing on the influence of a static annealing step, process temperature, and cooling atmosphere. **a**) Measured I_2D/_I_G_ ratio values. In-process annealing time is determined by the substrate velocity. **b**) SEM images indicating graphene coverage and Cu grain size at various reactor temperatures, where the same temperature is maintained in both zones, and all substrates are processed at 25 mm/min.

Last, we sought to identify principles for improved R2R production of graphene using the CTCVD system. It is understood that annealing prior to carbon exposure improves the viability of the Cu substrate for high-quality graphene growth by reducing surface oxides, healing surface defects on Cu, and by promoting Cu grain growth^29^,^30^,^35^. While it appears (based on the rate and degree of surface charging during SEM imaging) that the oxide layer on the as received Cu substrate is removed during annealing, qualitatively this does not change comparing annealing speeds of 500 mm/min to 25 mm/min (see Supporting Information, Fig. S9). To explore the utility of a more thorough foil pre-treatment step, Cu substrates were annealed in place for three hours at 1010 ^o^C and then the carbon source was turned on in the growth zone, and translation motion was started for graphene growth. We compared speeds of 25 and 125 mm/min, at which we had previously observed full graphene coverage on most Cu grains, and disconnected nanoscale graphene domains, respectively in the baseline study. With the extended pre-annealing step, a significant increase in the graphene I_2D_/I_G_ ratio to 1.39 was realized at 125 mm/min (Fig. 7a), as compared to the baseline process I_2D_/I_G_ ratio of 0.74 (Fig. 3). However, the I_2D_/I_G_ ratio for the 25 mm/min sample remained roughly the same at 1.48 as compared to the baseline value of 1.57. Representative Raman spectra are shown in Fig. S10 (see Supporting Information). Finally, previous studies have attributed the increase in the I_2D_/I_G_ ratio after similar annealing treatment to Cu grain growth^40^; however, on our samples we did not notice significant grain growth after annealing. Therefore, we attribute the higher quality to improvement of the foil surface chemistry and removal of surface defects.

We also anticipated that an increase in the reactor temperature would improve graphene quality, and coverage, by further promoting Cu grain growth and surface defect reduction during annealing^33^, and by increasing the kinetics of the formation of sp^2^ carbon on the surface^22^,^25^,^41^. To evaluate this hypothesis, experiments were performed at 25 mm/min and set point temperatures of 1000 °C, 1025 °C and 1045 °C (both annealing and growth processes were at these temperatures). At both 1000 °C and 1025 °C smaller graphene domains and incomplete coverage are observed by SEM; however, at 1045 °C there was a significant increase in graphene coverage and domain size (Fig. 7b), which is concurrent with the changes in the Raman I_2D_/I_G_ ratios (Fig. 7a). Therefore, we find that increasing the reactor temperature along with pre-treatment of the Cu foil (which could be performed in a batch process) results in much higher quality graphene, having greater domain size and coverage as measured by Raman spectroscopy and SEM on Cu.

Further, it is known that polycyclic aromatic hydrocarbons (PAHs) form in the CVD environment^42^, and that during cooling the PAHs deposit on exposed surfaces such as the tube walls and the Cu foil passing through the exit of the furnace. Deposition of these larger hydrocarbons could introduce amorphous carbon on the Cu and graphene surfaces, reducing the apparent quality of the film as examined through Raman spectroscopy. Coating of PAHs could also hinder the formation of graphene through carbon precipitation during cooling, as previously observed on Cu substrates^29^. To evaluate this potential variable, samples were run at 125 mm/min, and after a period of continuous operation, foil motion and the C_2_H_4_ flow was terminated (H_2_ and He were maintained). After 20 seconds of only He and H_2_ flowing in the reactor (enough time to allow the hydrocarbon to be completely removed), the system was cooled according to the normal procedure. On the region that was cooled within the growth zone of the furnace while exposed only to He/H_2_ flow, we observed a moderate average increase in the I_2D_/I_G_ ratio from 0.74 to 1.37 (Fig. 7a). As a result we believe that controlling the cooling atmosphere can also improve the performance of R2R graphene growth on the Cu substrate. However, we also note increased duration exposure to He/H_2_ atmosphere at elevated temperatures can etch the graphene^43^.

## Discussion

Beyond the characteristics of graphene achieved in this study, the design principles embodied by the CTCVD system are important to establish high-rate, high-quality continuous manufacturing of graphene on metal foils. First, because the cross-sectional area (*i.e.* the annular region) of the CTCVD design is significantly less than a standard circular tube, feedstock gas consumption can be reduced by greater than 90% while for an equivalent average velocity. Efficient conversion and consumption of feedstock is essential from a cost and environmental standpoint when considering manufacturing at scale^44^,^45^.

Second, graphene characteristics can be tuned *via* the reactor dimensions and process parameters. For example, replacement of the present single-zone heater tube furnace with a three-zone heater furnace would allow maintenance of separate thermal zones for annealing, graphene nucleation (*i.e.*, at the downstream injection point), and graphene growth. The conditions for each zone can therefore be optimized, along with the foil characteristics, to achieve the desired coverage and uniformity of graphene on the foil. The relative residence time can be specified in each zone by the length of each zone and by the single helical pitch of the wrap. The observed inverse relationship between graphene coverage and process rate (*i.e.*, substrate velocity) suggests that improved graphene coverage at high rate can be achieved by increasing the furnace length. Further, the same downstream injection principle could be implemented for atmospheric pressure CVD processing, along with custom rectangular (flat) tubes that could have one or more substrates translating on each inner tube surface. Each of these characteristics enables the bench-top CTCVD system to be scaled for graphene production at high rate and quality.

Nevertheless, there will be practical limits to the process conditions that can be implemented. For example, the maximum temperature will be limited by carbon pyrolysis and softening of the Cu substrate (which causes it to stretch and break under tension below its melting temperature). Also, to establish a third distinct gas zone for cooling of the substrate, a third gas mixture could be injected upstream from the far end of the annular gap, and issued outward through the inner tube while reversing direction and rapidly combining with the hydrocarbon flow away from the substrate.

Improvements in foil manufacturing and pre-treatment to reduce surface roughness and microstructure are also very important to improve the quality of the graphene produced. Also, for many applications where graphene must be transferred to a secondary substrate such as PET, it will be important to integrate continuous transfer and lamination methods^13^,^46^ whose throughput can match the roll-to-roll CVD parameters. Through the use of a large end chamber, the transfer and lamination processes could be housed adjacent to the CVD system.

We also consider that the graphene growth process itself is what ultimately limits the production rate. Initial modeling of the heat transfer to the Cu foil in the CTCVD system predicts that the foil can be heated to 1000 °C within 10 mm of furnace entry, at a speed exceeding 5 m/min. Therefore, discovery of methods to improve graphene nucleation and growth kinetics will not be limited by the thermal mass of the foil, and in fact contact between the foil and the inner tube significantly increases the heating rate compared to a freestanding foil. Based on present results, individual grains become fully covered within a residence time equivalent to 250 mm/min, which to our knowledge is the fastest rate yet reported in academic literature on R2R graphene growth.

In summary, the CTCVD design presents a scalable and modular approach to continuous production of thin films such as graphene on flexible substrates. While roll-to-roll synthesis of high-quality graphene is achieved in this study, the greater value of this work lies in the understanding of the process parameter space along with challenges presented by the characteristics of the metal substrate. In future work, integration of online metrology (*e.g., in situ* Raman spectroscopy) and real-time data analysis can help accelerate the identification of process conditions for improved and application-specific material characteristics. Wider availability of graphene in a continuous format will also be useful to advance transfer and patterning methods for 2-D device manufacturing.

## Methods

### Sample preparation

The desired length of 0.25" wide, 0.002" thick metal foil (www.metalribbon.com, Cu, 99.99% purity) was rinsed and manually wiped using Kimwipes soaked with acetone, and then wiped with Kimwipes soaked in isopropyl alcohol. The foil was anchored to the supply roller and wrapped onto the roller, and then the roller was manually loaded into the supply chamber. The foil was then wrapped around the inner tube and fed from the supply roller such that the helical path around the inner tube consisted of 1.5 revolutions between the supply and take-up rollers. Upon reaching the take-up reel, the downstream end of the foil was adhered to the take-up reel, and the take-up reel was rotated/advanced 90° (translating the foil ~50 mm) to ensure free motion of the foil through the system.

### Graphene growth

Heating was provided using a Lindberg Blue M Mini-Mite tube furnace, with a 25 mm OD quartz tube to serve as the outer tube (22 mm ID, 300 mm heated length). The inner tube used for all experiments was a 13 mm OD (10 mm ID) quartz tube, resulting in a tube-tube annular gap of 4.5 mm. Gas flow control (Aalborg MFCs) was dictated by the user through a custom LabView control interface. Prior to loading the foil for each experiment, the system was baked at a furnace temperature of 875 °C with a 500 sccm flow of dry air for 30 min. This step removed carbon deposits from the inner tube surfaces and created a consistent baseline starting condition for each R2R experiment. Additionally, after loading the foil for each experiment, the system was evacuated to 2 Torr while flowing He to remove the air that was introduced during the installation of the substrate material. The furnace was then heated to 1010 °C and a pressure of 4 Torr, with flows of 100 sccm H_2_ in both the inner tube and the tube gap. Next, foil translation was initiated at the desired velocity setpoint, and the gas flows were changed to 300 sccm H_2_ for the tube gap, and 10/315 sccm C_2_H_4_/H_2_ for the inner tube. Once the desired length of foil had been processed, the foil translation was stopped and the furnace was rapidly cooled by opening the furnace and applying a cold air stream across the reactor wall with a fan. This was done while maintaining the same gas flows until the furnace thermocouple reading dropped below 250 °C. The system was then purged with He for 10 min prior to removal of the processed foil. All gases used were supplied by Cryogenic Gases.

### Graphene transfer

The backside of the Cu foil was wet sanded with emery paper to remove the graphene layer on that side. The sample was then floated, graphene face up, in an aqueous solution of iron nitrate (50 mg/ml H_2_O) for 24 hours to etch the metal foil^10^, leaving behind the graphene on the solution surface. A SiO_2_ substrate was then carefully immersed in the solution and slowly removed such that the graphene was pinned to the SiO_2_ substrate by the meniscus during the substrate extraction. Following a rinse in deionized water, the sample was dried at 50 °C on a hot plate, in air, for 10 minutes.

### Characterization

Raman spectroscopy was conducted using a 532 nm laser (WITec Alpha 300R) on both as-grown and transferred graphene samples. Unless otherwise stated, the synthesized graphene was analyzed along the length (every 75 mm) and width (3 points, both edges and middle) of the substrate using Raman spectroscopy directly on the Cu foil. Spectroscopy measurements were collected using a 1 sec integration time, and background values were subtracted from the resulting data using a polynomial fit function. A Lorentzian peak-fitting algorithm was used along with a low-pass filter to generate spectra for further analysis. Two-dimensional Raman scans were conducted using the WITec Alpha 300 R and a 35 μm × 35 μm area of interest, 45 × 45 pixels, and an integration time of 0.5 sec for each data point. Optical microscopy was also performed on the WITec Alpha 300R system. FE-SEM secondary electron images were acquired using a Zeiss Supra55VP FESEM operated at 2–5 kV.

## Author Contributions

A.J.H. and E.S.P. conceptualized and designed the concentric tube CVD system, which was built by E.S.P. E.S.P. fabricated graphene films; E.S.P. and D.Q.M. performed optical microscopy, Raman spectroscopy and corresponding analysis; E.S.P., B.V. and S.W.P. performed scanning electron microscopy; E.S.P. and D.Q.M. performed graphene transfer; and A.J.H. supervised the research. E.S.P., D.Q.M. and A.J.H. wrote the manuscript. All authors discussed the results and reviewed and commented on the manuscript.

## Additional Information

**How to cite this article**: Polsen, E. S. *et al.* High-speed roll-to-roll manufacturing of graphene using a concentric tube CVD reactor. *Sci. Rep.* **5**, 10257; doi: 10.1038/srep10257 (2015).

## Supplementary Material

Supporting Information
