# Supplementary material for: High-speed roll-to-roll manufacturing of graphene using a concentric tube CVD reactor
Source: Sci Rep. 2015 May 21;5:10257. doi: 10.1038/srep10257 (PMC4440526; doi:10.1038/srep10257)
Supplement: Supporting Information [file srep10257-s1.pdf]

SUPPORTING INFORMATION for

High-speed roll-to-roll manufacturing of graphene using a concentric tube CVD reactor

*Erik S. Polsen[1,+], Daniel Q. McNerny[1], B. Viswanath [2,‡], Sebastian W. Pattinson[2], and A. John Hart[1,2]**

[1]Department of Mechanical Engineering, University of Michigan, 2350 Hayward St., Ann Arbor, MI 48109, United States.

[2]Department of Mechanical Engineering and Laboratory for Manufacturing and Productivity, Massachusetts Institute of Technology, Cambridge, MA, USA

[+] Current address: US Army TARDEC, RDTA-RTI-GSS, MS 263, 6501 E. 11 Mile Road, Warren, MI 48397-5000.

[‡] Current address: School of Engineering, Indian Institute of Technology Mandi, Himachal Pradesh, India

*CORRESPONDING AUTHOR: A. John Hart, ajhart@mit.edu, 617.324.7022

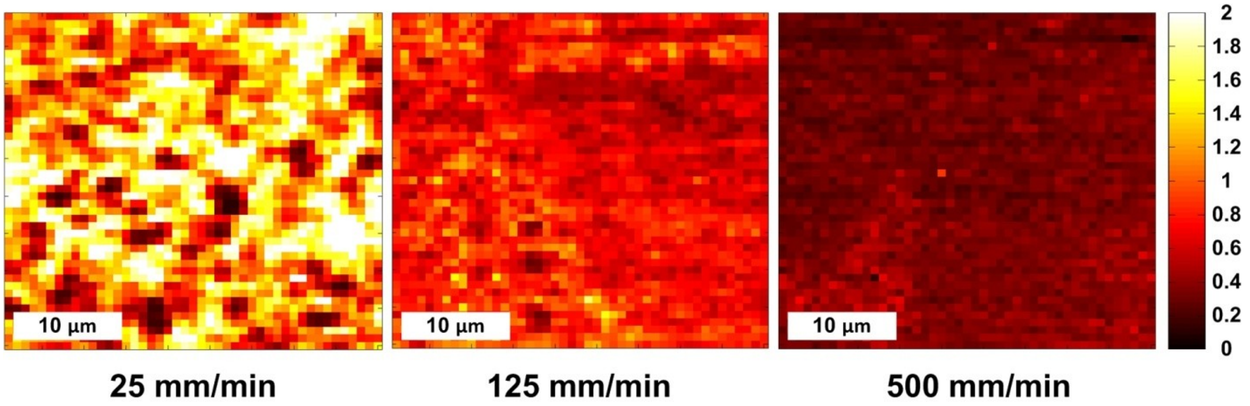

Figure S1. Two-dimensional Raman maps of I_{2D}/I_G ratio on Cu foil, processed at speeds noted above. The scans show that the graphene is fairly uniform across scans within individual Cu grains, along with the previously shown decrease in I_{2D}/I_G ratio with increased substrate velocity.

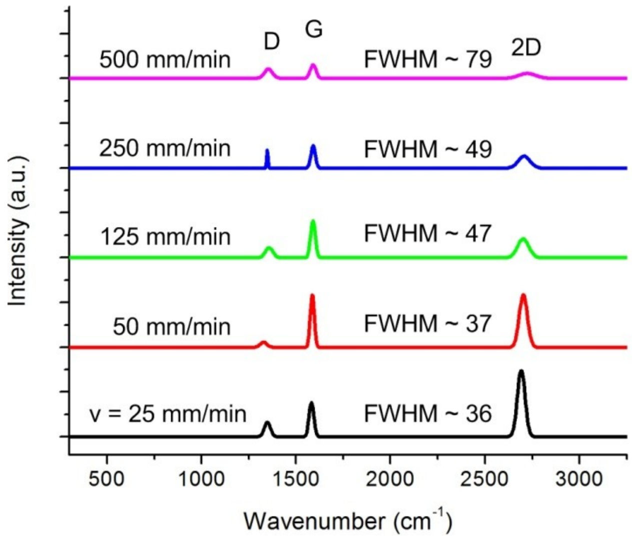

Figure S2. Graphene peak fits of Raman spectra. Examples of Raman spectra of graphene at various translation speeds after applying a Lorentzian multiple peak fit to the spectra and performing a complete background subtraction (see Methods). Full width half maximum (FWHM) values for the 2D peaks are also noted.

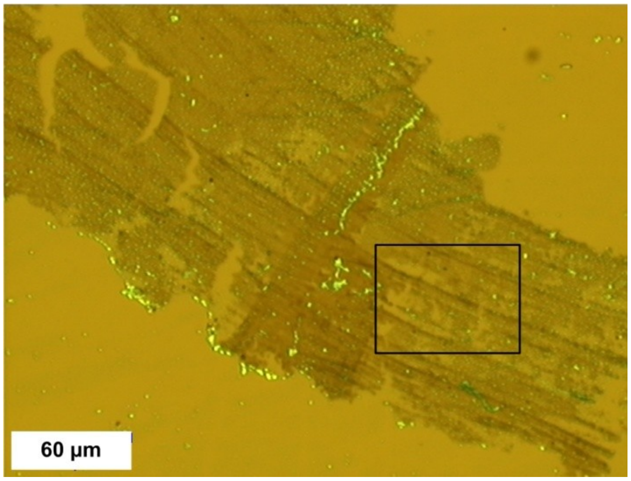

Figure S3. Optical image of graphene transferred to SiO_2. The box highlights an area where the individual nanoscale domains of graphene were washed away by the etching process and not transferred.

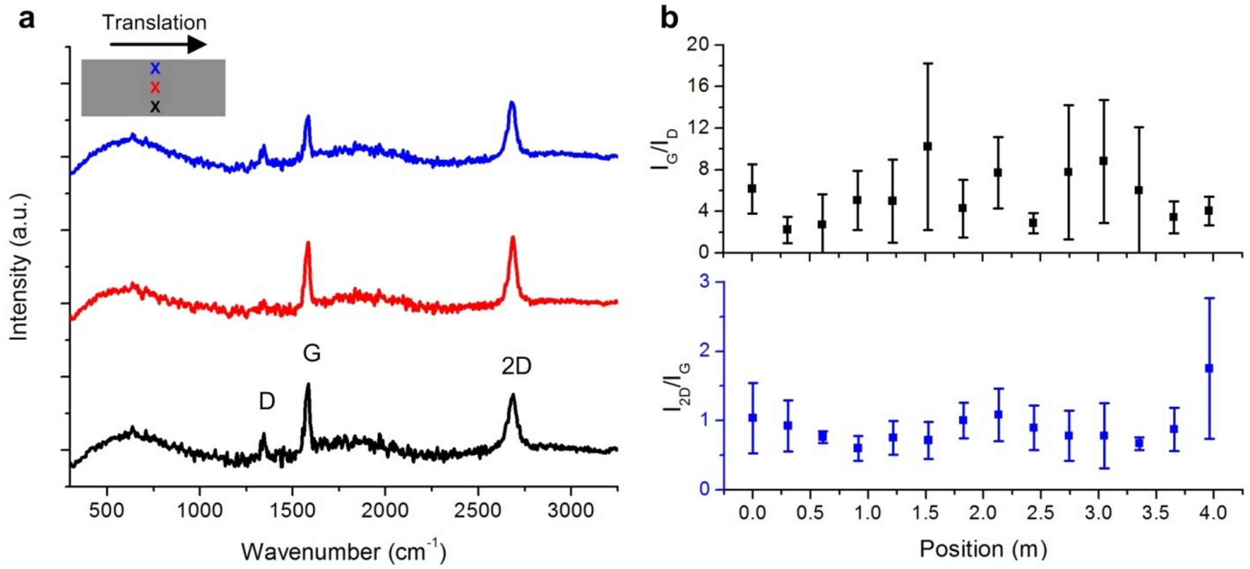

Figure S4. Assessment of graphene uniformity grown on Cu using the CTCVD system. a) Sample Raman spectra taken at three points along the width of the Cu foil (center and edges of the Cu foil as depicted by the inset diagram). b) Average I_G/I_D and I_{2D}/I_G values each representing the span across the width of the substrate, as in a), at 300 mm intervals along the length of the substrate.

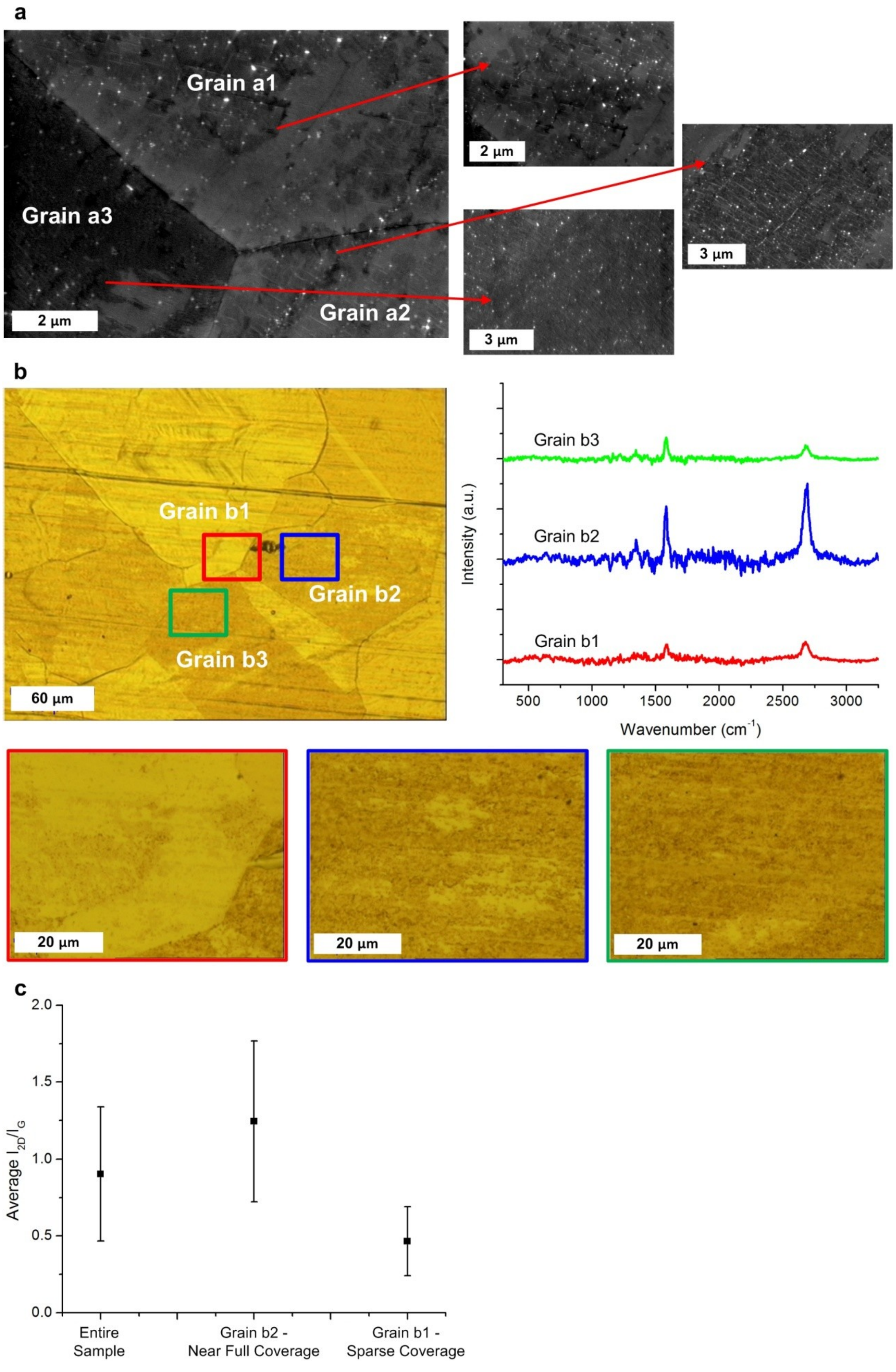

a
Grain a1
Grain a2
Grain a3
2 µm
2 µm
3 µm
3 µm
b
Grain b1
Grain b2
Grain b3
60 µm
Intensity (a.u.)
Wavenumber (cm^{-1})
500
1000
1500
2000
2500
3000
20 µm
20 µm
20 µm
c
Average I_{2D}/I_G
2.0
1.5
1.0
0.5
0.0
Entire Sample
Grain b2 - Near Full Coverage
Grain b1 - Sparse Coverage

Figure S5. Imaging of graphene growth differences on adjacent Cu grains. a) SEM image taken after graphene growth (75 mm/min) at the intersection between three Cu grains (left); contrast indicates difference in graphene coverage and morphology in each grain. b) Visible light microscopy of a similar three grain boundary, as shown in a), after graphene growth (25 mm/min). Corresponding Raman spectra (upper right) and magnified optical images (bottom) of the three neighboring Cu grains (highlighted in upper left image). c) Comparison of the average I_{2D}/I_G values across an entire sample with those on single grains of Cu that exhibited higher and lower quality graphene, possibly due to the difference in their orientation.

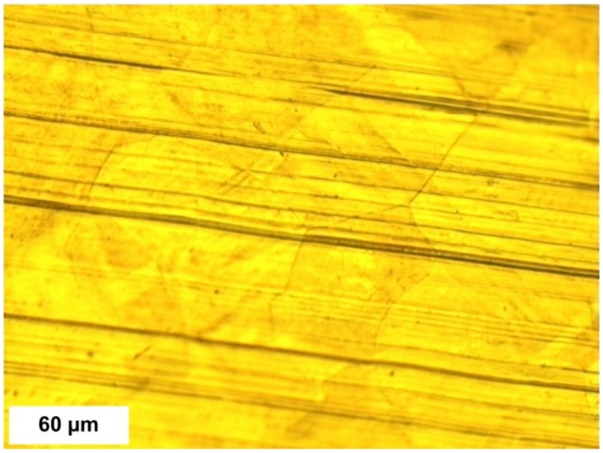

Figure S6. Example of surface defects and striations as seen by optical microscopy. The dark lines running the width of the image are the surface striations in the Cu substrate, and the small dark dots are indents in the surface.

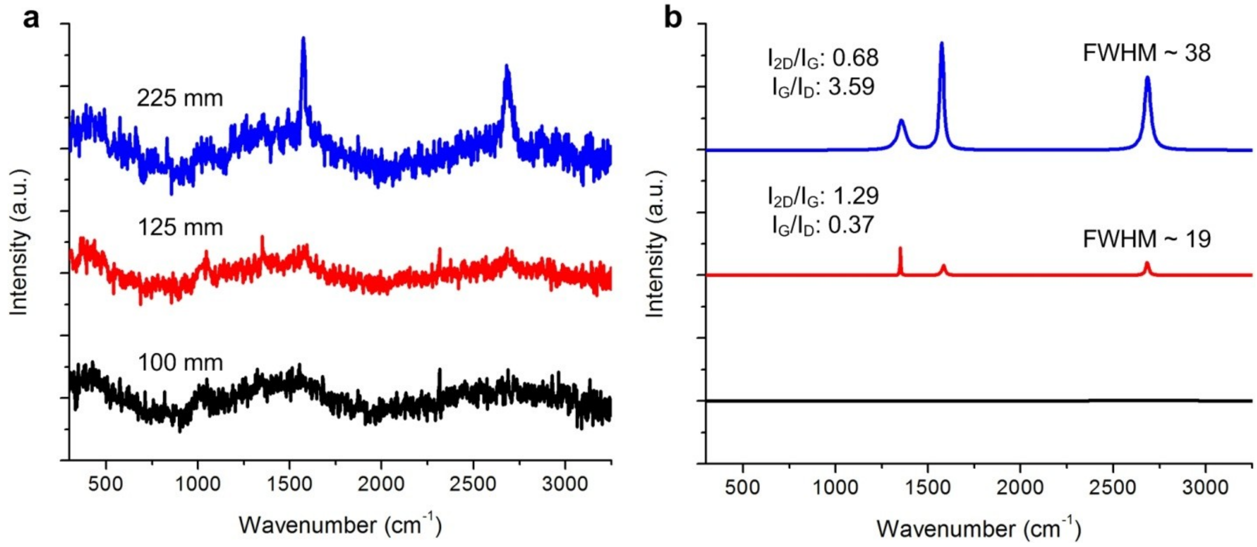

Figure S7. Raman spectra of sequential reduction of Cu, graphene nucleation, and graphene growth as determined by the position of the foil along the CTCVD reactor. a) Average Raman spectra for the three positions of interest (100, 125 and 225 mm) where a polynomial fit of the background signal was subtracted from the raw Raman spectra. Indicative G and 2D peaks are not present during the reduction of Cu (100 mm). However, during initial stages of nucleation (125 mm) the G and 2D peaks begin to emerge, and are apparent where the graphene has coalesced (225 mm). b) Raman spectra from a) after applying a Lorentzian multiple peak fit to the spectra and performing a complete background subtraction (see Methods). Full width half maximum (FWHM) values for the 2D peaks, and I_{2D}/I_G and I_G/I_D ratios are shown for the 125 and 225 mm samples.

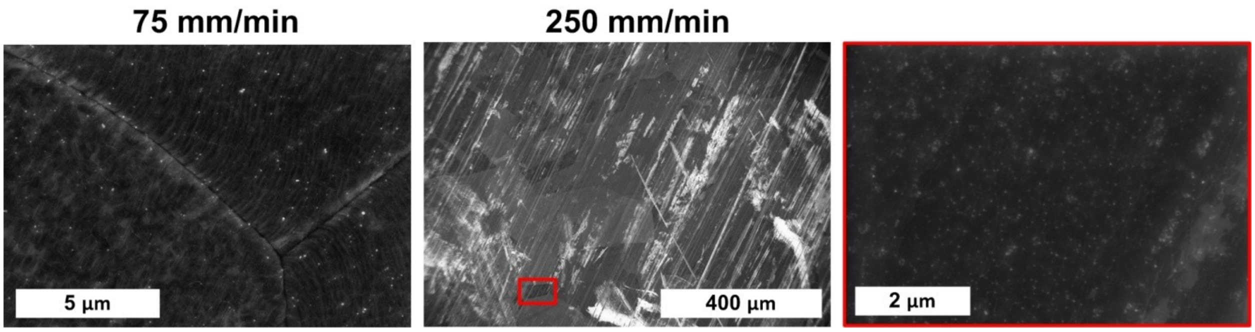

Figure S8. SEM images of typical full graphene coverage at 75 mm/min (left), and an example Cu grain with near full graphene at 250 mm/min (middle, right).

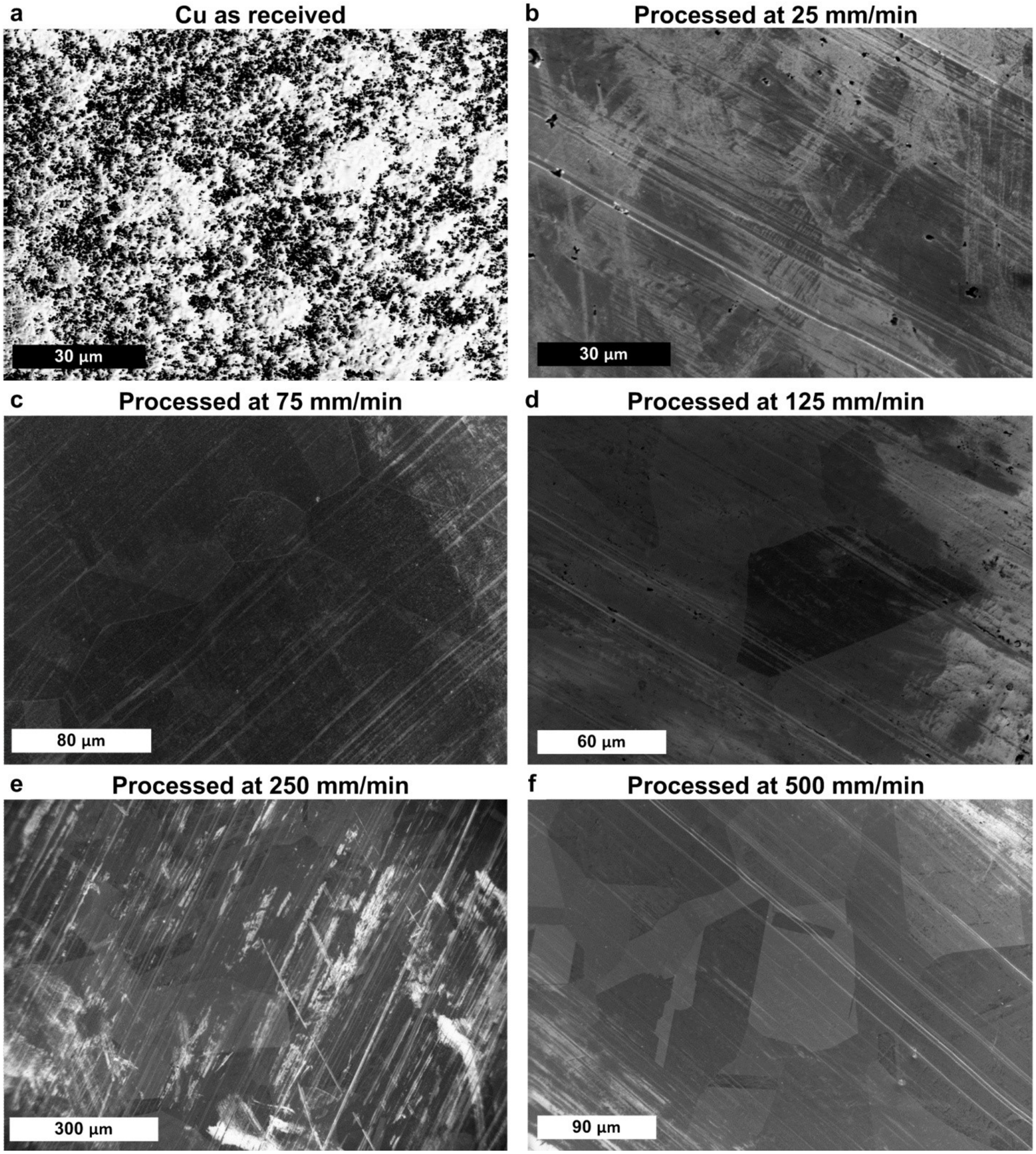

Figure S9. H_2 annealing at various substrate translation speeds influences the microstructural development of the Cu foil. a) SEM image of a section of the Cu that was not exposed to the annealing zone, and thus has surface oxide present. b – f) SEM images of Cu that was processed

in the CTCVD system (annealing and growth zones) at translation speeds of 25, 75, 125, 250 and 500 mm/min respectively.

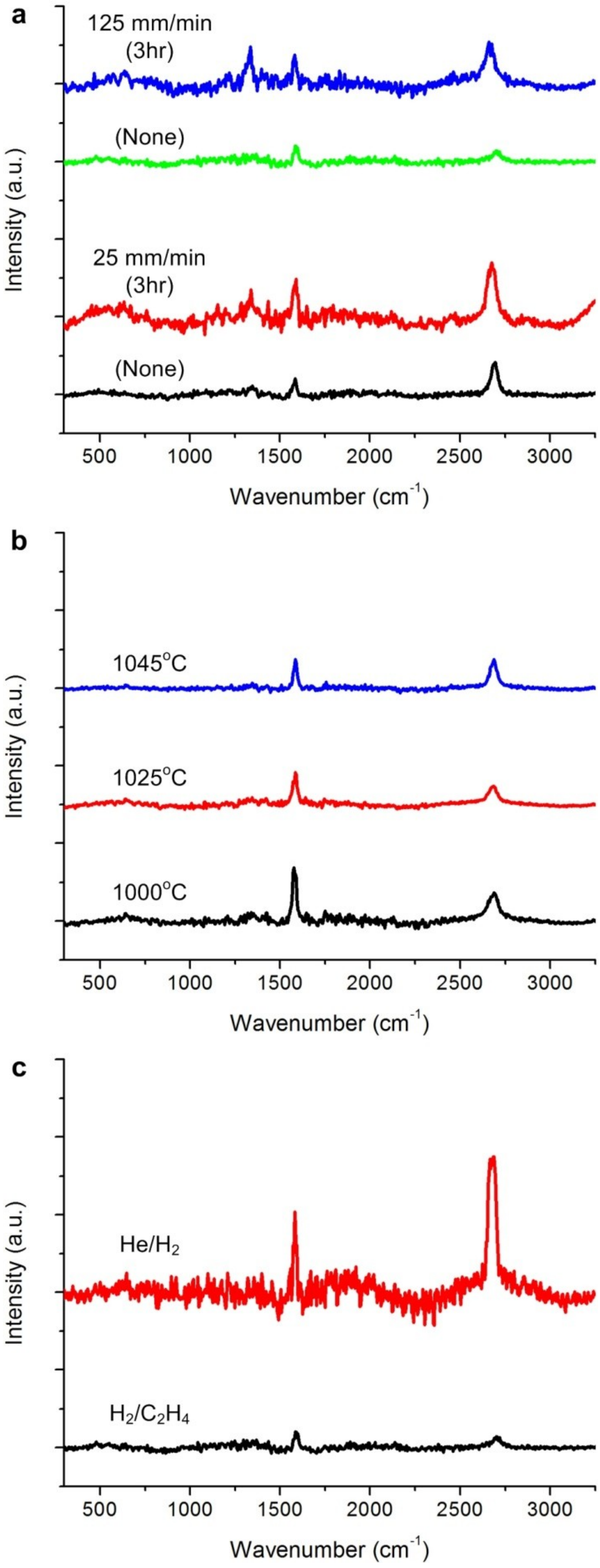
a
125 mm/min
(3hr)
(None)
25 mm/min
(3hr)
(None)
Intensity (a.u.)
500
1000
1500
2000
2500
3000
Wavenumber (cm-1)
b
1045oC
1025oC
1000oC
Intensity (a.u.)
Wavenumber (cm-1)
c
He/H2
H2/C2H4
Intensity (a.u.)
Wavenumber (cm-1)

Figure S10. Raman analysis of graphene processed at various conditions in the parametric study. a) Influence of 3 hour static annealing of the Cu foil prior to CTCVD processing at 25 and 125 mm/min. b) Normal processing (no static anneal) at 25mm/min, at 1000^oC, 1025^oC and 1045^oC. c) Normal processing at 125 mm/min cooled with (H_2/C_2H_4) and without (He/H_2) the carbon precursor products.
